# Evaluation of global and regional myocardial work in hypertrophic cardiomyopathy patients by left ventricular pressure-strain loop

**DOI:** 10.1186/s12872-023-03519-x

**Published:** 2023-09-27

**Authors:** Chengwei Xiao, Xuebing Zhao, Lijuan Sun, Fang Zhang

**Affiliations:** 1 Hebei Medical University, Shijiazhuang, China; 2 Department of Ultrasound, First Hospital of Qinhuangdao, Qinhuangdao, China

**Keywords:** Hypertrophic cardiomyopathy, Left ventricle, Pressure-strain loop, Echocardiography, Myocardial work

## Abstract

**Objective:**

This study aimed to investigate the value of left ventricular (LV) press-strain loop (PSL) in evaluating global and regional myocardial work (MW) in hypertrophic cardiomyopathy (HCM) patients.

**Methods:**

A total of 30 HCM patients with interventricular septum hypertrophy (HCM group) and 35 healthy subjects (control group) were selected from First Hospital of Qinhuangdao. The general clinical data and conventional ultrasound parameters of two groups were acquired. The MW parameters were analyzed using LV PSL. The regional MW parameters in the HCM group were compared between ventricular septum and the free walls of left ventricle.

**Results:**

The epicardial adipose tissue thickness of the HCM group was significantly greater than that of the control group (*P* < 0.05). Global work efficiency was significantly reduced, while global wasted work was increased in patients with HCM compared with controls (all *P* < 0.05). The HCM group was compared in the group, to be specific, in the HCM group, the work index, the work efficiency, and the longitudinal strain on the interventricular septum were lower than those on the free wall (all *P* < 0.05).

**Conclusion:**

PSL is more effective than LVEF in assessing left ventricular systolic function in HCM and is able to quantify regional myocardial work in the ventricular septum in HCM patients with preserved LVEF, suggesting a novel idea for clinical diagnosis and assessment.

## Introduction

Hypertrophic cardiomyopathy (HCM) refers to a common inherited cardiomyopathy and a common cause of sudden death in young adults with a prevalence of nearly 1 in 500. HCM is attributed to over 1400 mutations in 11 or more genes encoding cardiac sarcomere proteins [1, 2]. HCM is defined as a type of cardiac disease characterized by left ventricular hypertrophy (LVH) that arises from variations of sarcomerin-coding genes (or sarcomerin-associated genes) or unknown genetic etiology, except for conditions with clear evidence of other cardiac, systemic or metabolic diseases triggering LVH [3]. With the increase of the risk of sudden death over the past few years, more insights have been gained into this disease [4]. If the disease can be diagnosed at an early stage, and the degree of myocardial damage can be accurately evaluated, various methods can be employed as soon as possible to delay the course of the disease, prolong life, and improve patients’ quality of life, which comprise implantable defibrillator to prevent sudden death, as well as drug and surgical resection (or Percutaneous alcohol septal ablation) for relieving outflow obstruction, mitigating heart failure symptoms, controlling atrial fibrillation, and preventing embolic stroke [1].

Conventional echocardiography is capable of diagnosing the disease at the early stage, whereas it cannot accurately evaluate the myocardial damage due to the compensation of the myocardium. Left ventricular (LV) pressure-strain loop (PSL) refers to a novel method for the noninvasive evaluation of myocardial work (MW). Brachial artery blood pressure is adopted to replace LV pressure based on two-dimensional speckle tracking technology (2D-STI), such that the combination of LV strain and pressure parameters is capable of reducing the effect of afterload on myocardial strain, so as to evaluate the changes of MW more accurately [5].

Existing research has shown that broad the late gadolinium enhancement (LGE) from quantitative contrast-enhanced cardiovascular magnetic resonance imaging (MRI) measurements provides additional information to evaluate the risk of sudden cardiac death events in HCM patients, especially those judged to be at low risk [6]. Despite normal left ventricular ejection fraction (LVEF), global constructive work (GCW) is significantly reduced in HCM and is correlated with left ventricular fibrosis as evaluated by LGE [7]. Echocardiography and blood pressure measurement were used to noninvasively evaluate the reduced MW in patients with non-obstructive HCM. It is correlated with maximal LV wall thickness and is significantly correlated with worse long-term outcomes [8].

The aim of this study was to evaluate the global MW impairment in HCM patients with preserved LVEF using PSL technology and compare the interventricular septum and free wall regional myocardial work parameters in patients with HCM group, and further lay a theoretical basis for clinical intervention.

## Methods

### Study population

A total of 36 HCM patients were collected in this study, among which 2 patients were excluded due to combined apical hypertrophy, 2 patients were excluded due to poor image quality, and 2 patients had incomplete data. Finally, a total of 30 HCM patients with interventricular septum thickening ≥ 15mm and excluding inter ventricular septum thickening for other reasons were selected. The inclusion criteria conformed to the 2020 AHA/ACC guidelines for the Diagnosis and treatment of Hypertrophic Cardiomyopathy [3]diagnostic criteria for HCM: ① In the absence of other clear causes of myocardial hypertrophy, 2D echocardiography or cardiovascular magnetic resonance imaging (CMR) indicated a maximum end-diastolic wall thickness of 15 mm or more in any part of the LV. ② Besides, HCM can be diagnosed with ventricular wall hypertrophy (13–14 mm) when there is a family history of HCM or the gene test is positive. The exclusion criteria of patients included those with myocardial hypertrophy arising from hypertension, left ventricular outflow tract obstruction (Peak gradient > 30mmHg), diabetes, renal failure, valvular heart disease, myocardial amyloidosis, myocardial infarction, congenital heart disease, chronic obstructive pulmonary disease, arrhythmia, heart failure, and poor image quality. In the HCM group, approximately 60% of the patients had unknown HCM at the outpatient physical examination and approximately 40% had known HCM. All patients had no clinical symptoms and signs and LVEF was in the normal range, i.e., HCM patients with preserved LVEF.

A total of 35 healthy subjects were assigned to the control group. No cardiovascular disease and other organic diseases were confirmed through medical history, physical examination, echocardiography and laboratory tests. This study protocol was approved by the Ethics Committee of the First Hospital of Qinhuangdao (2021Q082).

### Echocardiography

2D transthoracic echocardiography was performed using experienced sonographers based on GE Vivid E95 ultrasonic diagnostic instrument equipped with M5S probe and Echo PAC (PC version:203) workstation.

The body height and the weight were input when the information was entered, and the body surface area (BSA) was automatically calculated using the system. Moreover, left atrial diameter (LAD), left ventricular end-diastolic dimension (LVEDd), LVEF, interventricular septum thickness at end-diastole (IVSd), left ventricular posterior wall thickness at end-diastole (LVPWd), left atrial volume (LAV) and epicardial adipose tissue (EAT) thickness were examined, and left ventricular mass index (LVMI) was determined. EAT thickness was measured perpendicular to the free wall of the right ventricle on the parasternal long axis at the end of diastole [9]. LV-EDV and LV-ESV were measured using the biplane modified Simpson’s method [10]. The formula to calculate LVMI [11]: LVMI (g/m2) = 0.8{1.04[([LV end‐diastolic dimension (LVEDd) + end‐diastolic interventricular septal thickness (IVSd) + end‐diastolic posterior wall thickness (LVPWd)]^3^ − LVEDd^3^)]} + 0.6 and normalized to body surface area.

### Myocardial work

Russell [4] et al. have suggested that the brachial artery blood pressure examined by the cuff can be adopted to replace the LV pressure and combine with longitudinal strain, and adjust in accordance with the opening and closing time of the mitral valve and aortic valve, such that the MW can be obtained in a noninvasive manner. The original data images were copied to Echo PAC 203 workstation in DICOM format for image analysis. The left ventricular endocardium and wall contours of standard apical long-axis and two- and four-chamber views were automatically tracked using the system. The positions of the respective tracking point were carefully observed, and the segments with inaccurate identification were manually adjusted. Afterward, the left ventricular PSL analysis was performed, such that MW parameters were determined.

Global MW indicators are elucidated as follows: (1) global work index (GWI): the area of left ventricular PSL, which is the sum of myocardial work from mitral valve closure to mitral valve opening; (2) GCW: the work performed by the myocardium during shortening systole or lengthening isovolumic diastole contributes to left ventricular ejection; (3) global wasted work (GWW): the work conducted by the myocardium when it elongates during systole or shorts during isovolumic diastole, hindering left ventricular ejection; (4) global work efficiency (GWE): GCW as a percentage of the sum of GCW and GWW, which reflects the efficiency of mechanical energy to perform work in the whole cardiac cycle. The mean values of longitudinal strain (LS), work index (WI), and work efficiency (WE) at the interventricular septum and free wall can be calculated in accordance with the eye plot of the 17-segment bull. According to the 17-segment bull's eye map, the regional MW parameters of the interventricular septum were the average values of the 5 segments, including antero-septal basal, septal basal, antero-septal mid-LV, septal mid-LV and septal apical segments. And the average values of the other 12 segments were the regional MW parameters of the free wall.

### Statistical analysis

Continuous variables are expressed as mean ± SD when normally distributed, or they are expressed as median (interquartile range [IQR]) when not normally distributed. Categorical variables are expressed as absolute numbers and percentages. Differences in clinical and echocardiographic characteristics between HCM patients and control subjects were compared through Student’s t test, the Mann–Whitney U test, as appropriate. The correlations of CW with other clinical and echocardiographic parameters were evaluated using Pearson’s method and Spearman’s method in terms of continuous normally distributed and ordinal and continuous non-normally distributed parameters, respectively. Intraclass correlation coefficients were determined for inter-observer and intra-observer agreement in 10 randomly selected patients to evaluate reproducibility. Statistical analysis was conducted using SPSS version 26.0 (IBM SPSS Statistics, version 26.0). *P* < 0.05 indicated a difference that achieved statistical significance.

## Results

### Study population

The study population comprised 30 HCM patients (mean50 (20) years; 66.7% men) and 35 healthy subjects (mean44 (18) years; 71.4% men) (Table 1). No significant difference was identified in age, sex, height, weight, body mass index (BMI), BSA, heart rate, systolic blood pressure and diastolic blood pressure between the two groups.

**Table 1:** The general clinical of patients with HCM and control subject

Clinical characteristics	Control group (*n* = 35)	HCM group (*n* = 30)	*P*-value
Age (year)	44(18)	50(20)	0.209
Male (%)	25(71.4)	20(66.7)	0.681
Height (m)	1.70 ± 0.07	1.69 ± 0.09	0.354
Weight (kg)	74 ± 13	76 ± 16	0.598
BMI (kg/m^2^)	25.2 ± 2.9	26.4 ± 3.8	0.162
BSA (m^2^)	1.86 ± 0.18	1.86 ± 0.23	0.972
Heart rate (beats/min)	71(10)	73(21)	0.797
Systolic BP (mmHg)	120(20)	126(23)	0.071
Diastolic BP (mmHg)	80(6)	79(15)	0.353

Data are expressed as mean ± SD, number (percentage), or median (interquartile range)

Significantly different (*P* < 0.05) compared with the control subjects

*BMI* Body mass index, *BSA* Body surface area, *BP* Blood pressure

### Echocardiography

Compared with the control group, the LAD, IVSd, and LVPWd in the HCM group were significantly increased (all *P* < 0.001) (Table 2). Compared with the control group, the LVEDd, LVEDV, LVESV, and LAV were decreased (all *P* < 0.05). However, there was no significant difference in stroke volume (SV) and LVEF between the two groups. LVMI and EAT thickness were significantly increased compared with the control group (all *P* < 0.001).

**Table 2:** Conventional echocardiographic parameters of patients with HCM and control subjects

Echocardiographic parameters	Control group (*n* = 35)	HCM group (*n* = 30)	*P*-value
LAD (mm)	33 ± 3	38 ± 5	< 0.001
IVSd (mm)	8(2)	20(8)	< 0.001
LVEDd (mm)	47 ± 3	45 ± 5	0.002
LVPWd (mm)	8(0)	10(2)	< 0.001
LVEDV (ml)	106.0 ± 13.9	95.3 ± 19.5	0.015
LVESV (ml)	35.6 ± 5.8	31.3 ± 7.7	0.014
SV (ml)	70.5 ± 9.6	65.4 ± 16.1	0.135
LVEF (%)	66 ± 2	67 ± 3	0.650
LVMI (g/m^2^)	65.2 ± 11.2	162.0 ± 48.9	< 0.001
EAT thickness (mm)	1.68 ± 0.18	5.04 ± 1.27	< 0.001
LVA (ml)	41.9 ± 10.1	65.3 ± 18.8	< 0.001

Data are expressed as mean ± SD, number (percentage), or median (interquartile range)

Significantly different (*P* < 0 .05) compared with the control subjects

*LAD* Left atrial diameter, *IVSd* Interventricular septum thickness at end-diastole, *LVEDd* Left ventricular end-diastolic dimension, *LVPWd* Left ventricular posterior wall thickness at end-diastole, *LVEDV* Left ventricular end-diastolic volume, *LVESV* Left ventricular end-systolic volume, *SV* Stroke volume, *LVEF* Left ventricular ejection fraction, *LVMI* Left ventricular mass index, *EAT thickness* Epicardial adipose tissue thickness, *LAV* Left atrial volume

### Myocardial work parameters

GLS of the HCM group was significantly decreased, peak strain time dispersion (PSD) was significantly increased compared with the control group (Table 3). GWI was significantly decreased as compared with that of the control group. GCW significantly declined. However, GWW was significantly increased compared with the control group. Accordingly, GWE was significantly lower than that in the control group.

**Table 3:** GLS and MW parameters of patients with HCM and control subjects

parameters	Control group (*n* = 35)	HCM group(*n* = 30)	*P*-value
GWI (mmHg%)	1649.6 ± 217.7	1134.6 ± 450.8	< 0.001
GCW (mmHg%)	2012.6 ± 257.6	1394.6 ± 463.7	< 0.001
GWW (mmHg%)	93(74)	178(85)	< 0.001
GWE (%)	95(4)	84(9)	< 0.001
GLS (-%)	17.6(2.0)	12.0(6.6)	< 0.001
PSD (ms)	43.5(15.8)	123.1(52.3)	< 0.001

Data are expressed as mean ± SD or median (interquartile range)

Significantly different (*P* < 0 .05) compared with the control subjects

*GWI* Global work index, *GCW* Global constructive work, *GWW* Global wasted work, *GWE* Global work efficiency, *GLS* Global longitudinal strain, *PSD* Peak strain time dispersion

In the eye plot of the 17-segment bull, the LS of the interventricular septum was significantly lower than that of the free wall, and the WI of the interventricular septum was significantly lower than that of the free wall (Table 4). In comparison with the free wall, WE in the interventricular septum significantly declined.

**Table 4:** Regional MW parameters of ventricular septum and free wall in HCM group

parameters	Ventricular septum (*n* = 30)	Free wall (*n* = 30)	*P*-value
WI (mmHg%)	887.2 ± 387.2	1275.9 ± 517.6	0.002
WE (%)	81 ± 8	87 ± 7	0.006
LS (-%)	8.9 ± 4.3	13.1 ± 4.5	0.001

Data are expressed as mean ± SD

Significantly different (*P* < 0 .05) compared with the control subjects

*WI* Work index, *WE* Work efficiency, *LS* Longitudinal strain

### Myocardial work parameters were correlated with other parameters

GCW had a negative correlation with EAT thickness (*r* = -0.534, *P* < 0.001), IVSd (*r* = -0.699, *P* < 0.001), GLS (*r* = 0.737, *P* < 0.001), LVMI (*r* = -0.653, *P* < 0.001), PSD (*r* = -0.660, *P* < 0.001). GWI, GWW and GWE were also significant correlated with IVSd, EAT thickness, GLS, LVMI and PSD (Table 5). We also found that Regional WI and Regional WE showed a high correlated with IVSd, GLS, LVMI and PSD (Table 6).

**Table 5:** Correlation between global myocardial work parameters and other parameters of patients with HCM and control subjects

parameters	GWI		GCW		GWW		GWE	
	r	*P*-value	r	*P*-value	r	*P*-value	r	*P*-value
IVSd (mm)	-0.658	< 0.001	-0.699	< 0.001	0.496	< 0.001	-0.660	< 0.001
EAT thickness (mm)	–0.505	< 0.001	–0.534	< 0.001	0.470	< 0.001	–0.594	< 0.001
GLS (-%)	0.748	< 0.001	0.737	< 0.001	-0.514	< 0.001	0.707	< 0.001
LVMI (g/m^2^)	-0.612	< 0.001	-0.653	< 0.001	0.393	< 0.001	-0.690	< 0.001
PSD (ms)	-0.609	< 0.001	-0.660	< 0.001	0.659	< 0.001	-0.807	< 0.001
LAV (ml)	-0.223	0.074	-0.256	0.039	0.374	0.002	-0.352	0.004

Significantly different (*P* < 0 .05) compared with the control subjects

*IVSd* Interventricular septum thickness at end-diastole *EAT thickness* Epicardial adipose tissue thickness, *GLS* Global longitudinal strain, *LVMI* Left ventricular mass index, *PSD* Peak strain time dispersion, *LAV* Left atrial volume

**Table 6:** Correlation between regional myocardial work parameters and other parameters in HCM group

parameters	WI		WE	
	r	*P*-value	r	*P*-value
IVSd (mm)	-0.690	< 0.001	-0.540	0.002
GLS (-%)	0.515	0.004	0.491	0.006
LVMI (g/m^2^)	-0.385	0.036	-0.455	0.012
PSD (ms)	-0.384	0.036	-0.606	< 0.001

Significantly different (*P* < 0 .05) compared with the control subjects

*IVSd* Interventricular septum thickness at end-diastole, *GLS* Global longitudinal strain, *LVMI* Left ventricular mass index, *PSD* Peak strain time dispersion

### Intra-observer and inter-observer variability in myocardial work parameters

The correlation coefficients of the examined variables between different observers were as follows (Table 7). GWI 0.99 (95% CI,0.98–0.99), GCW 0.99(95% CI,0.97–0.99), GWE 0.82(95% CI,0.42–0.95), GWW 0.95(95% CI,0.81–0.98), and GLS 0.95(95% CI, 0.80–0.98) and PSD 0.93(95%CI,0.75–0.98). The correlation coefficients of the intra-observer examined variables for the respective parameter were as follows. GWI 0.99 (95% CI,0.98–0.99), GCW 0.99(95% CI,0.98–0.99), GWE 0.88(95% CI,0.60–0.97), GWW 0.84(95% CI,0.48–0.96) and GLS 0.97(95% CI,0.90–0.99) and PSD 0.84(95%CI,0.49–0.96).

**Table 7:** Intra-observer and inter-observer variability in myocardial work parameters

Parameters	Inter-observer variability			Intra-observer variability		
	ICC	95%CI	*P*-value	ICC	95%CI	*P*-value
GWI (mmHg%)	0.99	0.98–0.99	< 0.001	0.99	0.98–0.99	< 0.001
GCW (mmHg%)	0.99	0.97–0.99	< 0.001	0.99	0.98–0.99	< 0.001
GWW (mmHg%)	0.95	0.81–0.98	0.002	0.84	0.48–0.96	0.004
GWE (%)	0.82	0.42–0.95	0.004	0.88	0.60–0.97	0.003
GLS (-%)	0.95	0.80–0.98	0.002	0.97	0.90–0.99	< 0.001
PSD (ms)	0.93	0.75–0.98	0.002	0.84	0.49–0.96	0.004

Significantly different (*P* < 0 .05) compared with the control subjects

*GWI* Global work index, *GCW* Global constructive work, *GWW* Global wasted work, *GWE* Global work efficiency, *GLS* Global longitudinal strain, *PSD* Peak strain time dispersion

## Discussion

This study described the differences in global myocardial work parameters between HCM patients with ventricular septum hypertrophy and normal subjects, as well as the differences in regional work parameters of the ventricular septum compared with the free wall in the HCM group. The main findings of this study are elucidated as follows. ① Compared with normal subjects, the global myocardial work parameters GWI, GCW, GWE and GLS in HCM patients declined significantly, whereas EAT thickness, GWW and PSD were significantly increased. ② Regional myocardial work parameters WI, WE and LS of the septum were significantly lower than those of the free wall in HCM patients with hypertrophic ventricular septum. ③ Global MW indicators were significantly correlated with IVSD, EAT thickness, GLS, LVMI, PSD and LAV. WI、WE at the interventricular septum were significantly correlated with IVSD, GLS, LVMI and PSD.

Existing research has shown that GLS, as a new technique based on 2D speckle tracking, is more sensitive than LVEF at the early evaluation of left ventricular systolic function in HCM patients. Although GLS does not assess myocardial fibrosis as well as LGE in HCM patients, Almaas et al. [12] demonstrated that GLS was a more powerful tool for predicting arrhythmias than LGE. In the HCM group, all patients had no clinical symptoms and signs and LVEF was in the normal range, and there was no statistical significance in LVEF compared with the control group (*P* > 0.05). Compared with the control group, GLS in the HCM group significantly declined, consistent with the previous research results. When LVEF was normal, GLS could sensitively evaluate left ventricular systolic dysfunction at the early stage, and the thicker the ventricular wall, the lower the strain [13]. However, GLS does not consider the afterload factor, resulting in a large load dependence of the results. Left ventricular PSL is a new noninvasive index to evaluate left ventricular systolic performance. Based on 2D STI, it uses brachial artery blood pressure instead of left ventricular pressure, such that the combination of left ventricular strain and pressure parameters can reduce the effect of afterload on myocardial strain, such that the change of myocardial work can be more accurately evaluated. Russell [5] et al. proposed for the first time to replace the left ventricular pressure with a non-invasive method, and there was a good correlation between the area of PSL examined by the non-invasive method and the myocardial oxygen consumption detected by myocardial glucose metabolism. At present, PSL has been applied to a variety of cardiovascular diseases, such as aortic valve stenosis, coronary artery disease, and resynchronization therapy [14–17]. 

**Fig. 1:**
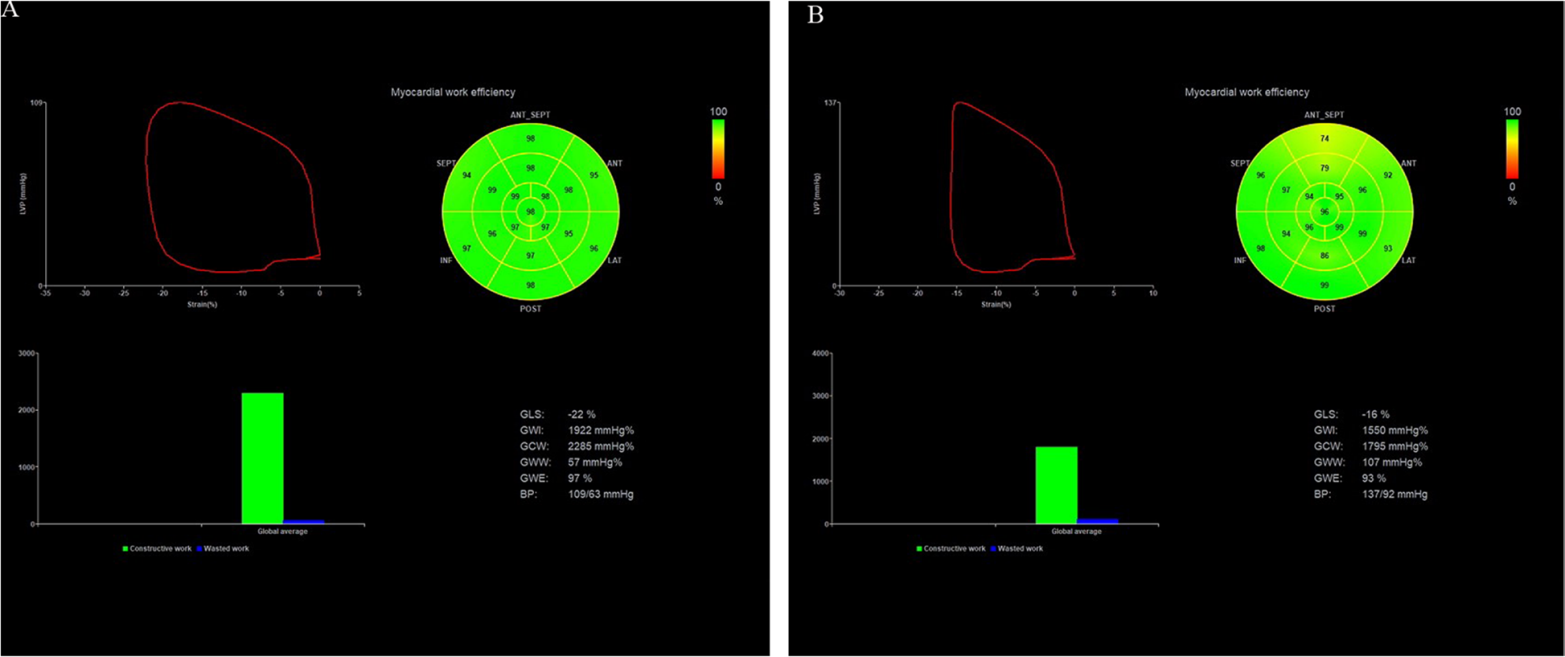
The pressure-strain loop, bull's eye plot of myocardial work efficiency, bar chart of GWW and GCW comparison, and myocardial work value in control group and HCM group were compared. **A** control group, (**B**) HCM group. *GWI*, global work index; *GCW*, global constructive work; *GWW*, global wasted work; *GWE*, global work efficiency; *GLS*, global longitudinal strain; *PSD*, peak strain time dispersion; *ANT*, Anterior; *ANT_SEPT*, antero-septal; *INF*, inferior; *LAT*, lateral; *POST*, posterior; *SEPT*, septal

**Fig. 2:**
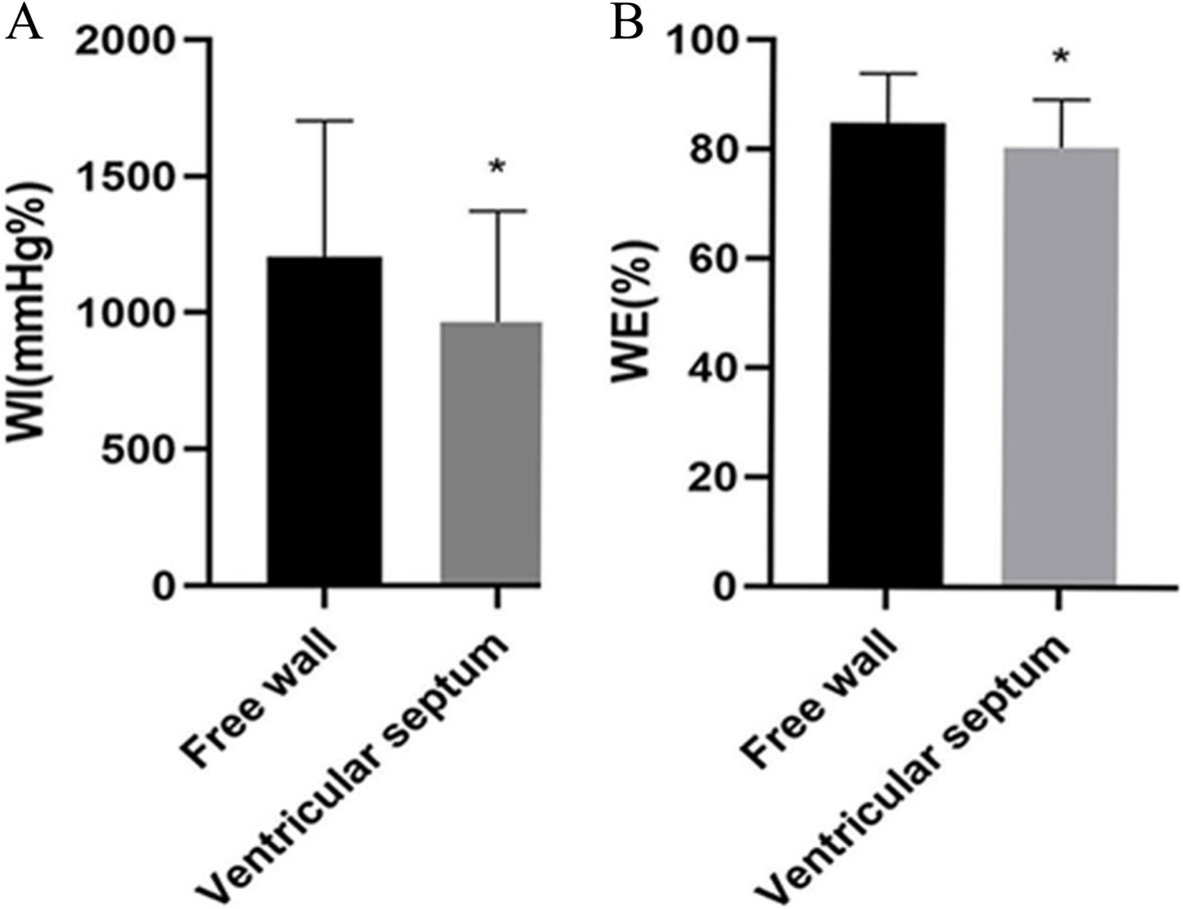
Comparison of regional WI (**A**) and WE (**B**) between ventricular septum and free wall in HCM group. (* means *P* < 0 .05 when ventricular septum compared with free wall). *WI*, work index; *WE*, work efficiency

Existing research has suggested that reduced myocardial work in non-obstructive HCM is correlated with maximal left ventricular wall thickness, and it shows a significant correlation with worse long-term prognosis [8]. In this study, GCW was significantly reduced in HCM patients, consistent with the findings of existing research (Fig. 1). Previous studies have proposed that GCW is the only predictor of left ventricular myocardial fibrosis in HCM patients [7], and a cut-off of ≤ 1550 mmHg% is significantly correlated with CMR results [18], so about 70% of HCM patients in this study had myocardial fibrosis. Given previous research, this study proposed a hypothesis that pathological changes (e.g., cardiomyocyte hypertrophy and myocardial fibrosis) may lead to reduced myocardial compliance and deformability, such that the time to peak strain can be extended, and myocardial work can be reduced. Moreover, the increase of GWW will increase myocardial oxygen consumption, thus exerting a certain effect on global and regional myocardial work. In this study, GLS and the overall myocardial work parameters (e.g., GCW, GWI, and GWE) were significantly decreased, whereas GWW and PSD were significantly increased in the HCM group, and GWW and PSD were more significantly increased with the gradual reduction of GCW, GWI, and GWE. Hypertrophy and fibrosis of cardiomyocytes resulted in impaired myocardial deformability, which was indicated by the decreased GLS. Impaired myocardial deformability led to decreased GCW and increased GWW, such that GWE was decreased. As depicted in Fig. 2, WI and We of the hypertrophic septal myocardium were significantly lower than those of the free wall, probably correlated with the hypertrophy of the thickened septal cardiomyocytes and myocardial fibrosis. Existing research has suggested that hypertrophy and fibrosis can result in damage in the shortened area of the myocardium in HCM patients [19], and then WW is increased, and WI and WE are decreased in the hypertrophic ventricular septum. We found that although the regional MW in the free wall of the HCM group was increased compared with the regional MW in the ventricular septal, it was still lower than that in the control group, which was similar to the results of Hiemstra et al. [8]. This phenomenon does not have the compensatory increase of free wall as in LBBB [20]. This may be related due to the different pathophysiological bases. HCM is the hypertrophy and fibrosis of cardiomyocytes caused by gene mutation, the adjacent ventricular wall is involved, and the regional work of the free wall is slightly reduced. The latter is a conduction disorder, due to intraventricular and interventricular dyssynchrony, the premature contraction of the ventricular septum, the lateral wall of the left ventricle stretches ahead of time, resulting in increased compensatory work of the left ventricular wall [21].

LVMI was significantly increased in the HCM group, showing a positive correlation with GWW and a negative correlation with regional WI and WE of the ventricular septum. Similar to the results of the regional myocardial work study, the regional strain of the interventricular septum was significantly lower than that of the free wall, with statistical significance (*P* < 0.05). The strain does not take into account the after-load factor, and the accuracy is not as good as in myocardial work. However, regional strain is still superior to general left ventricular longitudinal strain. Because studies have shown that the thicker the interventricular septum, the worse the strain, the more serious the systolic function is damaged, and the free wall also has myocardial systolic dysfunction [13]. The hypertrophy of cardiomyocytes in HCM patients led to myocardial fibrosis, and then the increase of LVMI resulted in the remodeling of the left ventricle and the increase of GWW. Existing research has suggested that in HCM patients, the degree of myocardial fibrosis is increased with the increase of myocardial hypertrophy, and myocardial fibrosis shows a positive correlation with LVMI [22]. Liu [23] et al. reported that LVMI is independently correlated with early left ventricular reverse remodeling.

We also found that LAV was significantly higher than that of the control group (*P* < 0.05). Although HCM patients in this study had no obvious clinical symptoms, LA function was also impaired in HCM patients with no symptoms or mild symptoms [24]. Moreover, we found that LAV was significantly correlated with GCW, GWW and GWE, considering that patients with HCM had both systolic and diastolic dysfunction.

In addition, this study also suggested that EAT thickness was significantly increased compared with normal subjects and positively correlated with GWW. Existing research has reported a significant correlation between increased EAT area and the occurrence of atrial fibrillation in HCM patients [25], and EAT thickness accumulation is correlated with worse hemodynamic and metabolic characteristics in HFpEF while affecting survival [26]. Accordingly, EAT thickness can serve as one of the evaluation parameters to gain insights into the risk of cardiac events in HCM patients. Jin [27] et al. reported that the thickness of the epicardial adipose tissue was greater in heart failure with preserved (HFpEF)than in reduced and mildly reduced ejection fraction patients. Moreover, the increase of the EAT thickness in HFpEF is correlated with the difference in LA function.

The correlation coefficients of the intra-observer and inter-observer variability in myocardial work Parameters were excellent.

In brief, when HCM patients have no clinical symptoms and signs and LVEF is still in the normal range, the global and regional myocardial work parameters obtained by non-invasive PSL can help clinical diagnosis and specific treatment plans reduce the risk of heart failure and sudden cardiac death as much as possible.

## Limitations

This study had a small sample size, and the sample size should be further expanded to confirm the research results. Brachial artery pressure was employed instead in this study. The left ventricular pressure was estimated, and the measurement of myocardial work parameters is dependent on the accuracy of blood pressure measurement, which may have some errors. PSL relies on 2D-STE, and the myocardial spots tracked by PSL are limited to the 2D plane rather than the 3D space, which has certain limitations. Consequently, a failure of some myocardial spots to be tracked may be caused, and the accuracy of the results may be affected.

## Conclusion

PSL, a novel non-invasive quantitative method to evaluate myocardial work, is capable of evaluating the global and regional myocardial work damage when HCM patients do not have any clinical symptoms and signs, and LVEF is still in the normal range, while laying a solid basis for clinical diagnosis and treatment. Thus, it is promising to a certain extent.

## Data Availability

The datasets used and/or analysed during the current study are available from the corresponding author on reasonable request.
